# Long-term outcomes and influencing factors following pediatric kidney transplantation: a single-center cohort study from China

**DOI:** 10.3389/fped.2025.1599111

**Published:** 2025-06-05

**Authors:** Junhao Yu, Xiaoju Sheng, Yuhong Li, Mingxing Sui, Jiazhao Fu, Li Zeng, Yanhua Li, Wenyu Zhao

**Affiliations:** Department of Organ Transplantation, Shanghai Changhai Hospital, Naval Medical University, Shanghai, China

**Keywords:** end-stage kidney disease, pediatric kidney transplantation, graft survival, long-term outcomes, near-final height

## Abstract

**Background:**

Kidney transplantation is recognized as the optimal treatment for end-stage kidney disease (ESKD) in children, which significantly improves growth delay, pubertal development, and social prognosis in pediatric patients. This study analyzed the long-term prognosis and influencing factors following pediatric kidney transplantation at our center.

**Methods:**

A total of 101 pediatric recipients who underwent kidney transplantation at our center were enrolled in this study. Post-transplant outcomes, including renal function, height development, pubertal progression, and social adaptation, were systematically analyzed.

**Results:**

The height-for-age Z-score (HAZ) significantly improved from −2.27 ± 1.64 at transplantation to −0.76 ± 1.13 after achieving post-transplant stability. The graft survival rates were 100% and 93.4% at 5 and 10 years post-transplantation, respectively, while patient survival rates remained 100% at both 5 and 10 years. At the last follow-up, the mean serum creatinine level was 83.85 ± 38.34 μmol/L, with an estimated glomerular filtration rate (eGFR) of 79.49 ± 27.25 ml/min/1.73 m^2^. Among school-aged recipients, 93.75% successfully returned to school, while only 33.3% of those who completed their education achieved employment. Among male adolescents (13 years, *n* = 43), 37 cases (86.0%) experienced spermarche, with a mean age of 14.5 years in prepubertal transplant recipients. In the female cohort (*n* = 45), 42 patients (12 years) reached puberty, demonstrating a mean menarche age of 12.5 years in prepubertal recipients vs. 13.2 years in postpubertal transplants (*P* > 0.05). Menstrual irregularities were observed in 8 cases, accounting for 19.51% of menstruating females.

**Conclusion:**

This study demonstrates significant improvements in height development, pubertal progression, and social adaptation following kidney transplantation in pediatric recipients. While recipient gender, pre-transplant dialysis modality, and dialysis duration showed no significant impact on near-final height (NFH), both transplantation age and height at transplantation significantly influenced NFH attainment. These findings emphasize that early transplantation and maintaining optimal graft function are crucial for ameliorating growth delay and pubertal development, while also positively influencing long-term social outcomes in pediatric transplant recipients.

## 1 Introduction

Poor long-term prognosis remains a significant concern in pediatric patients with chronic kidney disease (CKD). Kidney transplantation, recognized as the optimal treatment for end-stage kidney disease (ESKD) in children, has been shown to significantly improve growth and developmental, enhance both physical and psychological development, and promote social-psychological well-being, ultimately leading to improved quality of life and survival rates in this population (1). Prolonged dialysis in children with renal failure not only adversely affects growth and pubertal development but also significantly impacts long-term social outcomes, substantially increasing the risk of negative social and occupational consequences (2). In the past, the development of pediatric kidney transplantation in China has been hindered by the persistent shortage of pediatric donor organs. Our center pioneered the pediatric-to-pediatric (PTP) allocation model and has systematically validated its feasibility and clinical efficacy (3). Following the 2018 revision of China's kidney allocation policy, which prioritized pediatric recipients for pediatric donor kidneys, the field of pediatric kidney transplantation has developed rapidly in China.

Currently, significant improvements in transplantation success rates have been achieved through advancements in surgical techniques, optimization of immunosuppressive therapies, and enhanced comprehensive pre-transplant evaluation and perioperative management (4). While short-term prognosis of pediatric kidney transplantation has shown significant improvement, there remains substantial room for enhancing long-term graft survival. Studies have identified that all pediatric recipients undergo a high-risk period for graft failure during adolescence. This high-risk period is associated with multiple contributing factors, including enhanced immune reactivity, decreased medication adherence, and the transition from parental supervision to self-management, all of which collectively contribute to suboptimal long-term graft survival rates in pediatric recipients (1, 5, 6).

Given the relatively late initiation of pediatric kidney transplantation in China, there is a notable scarcity of research and data on long-term outcomes among pediatric recipients. As the pioneering transplant center in China to implement pediatric-to-pediatric kidney transplantation, our institution conducted a retrospective analysis of 101 pediatric recipients who received transplants from young pediatric donors. This study comprehensively evaluated post-transplant growth and development, pubertal progression, educational and occupational status. The detailed findings are presented below.

## 2 Methods

This retrospective study analyzed pediatric recipients who underwent kidney transplantation from 2013 to 2021 at Shanghai Changhai Hospital, Shanghai, China. Study data were systematically collected using a dual approach: (1) Targeted queries for patient basic information in our center's electronic medical record system. (2) The follow-up information of patients was collected by means of structured questionnaires. The research information includes donor information, recipient demographic characteristics, transplantation modality, primary disease, pre-transplant dialysis method and duration, graft function, recipient/graft survival rates, complication profiles, and the age of spermatorrhea in male patients and the age of menarche in female patients. Social outcome assessments included educational attainment and employment status.

The inclusion criteria were: (1) age <18 years at the time of transplantation; (2) minimum follow-up duration of 3 years; (3) no recombinant human growth hormone (rhGH) treatment during the follow-up period.

The final follow-up data included height measurements (in centimeters) and serum creatinine levels (Scr) for all patients. The estimated glomerular filtration rate (eGFR) was calculated using the creatinine-based Schwartz formula (7), this equation is applicable for children and adolescents aged 2 to 18 years. Growth evaluation methodology: The height-for-age z-score (HAZ) was utilized for assessment, calculated as (measured height—standard height for age and gender)/standard deviation. Height standards were referenced to the WHO 2007 Growth curve (8). Growth status was determined based on z-score values: normal growth was defined as |Z| ≤ 2, while growth delay was identified when Z < −2. As bone age assessment was not performed, near-final height (NFH) was defined as annual height gain < 0.5 cm (9). The assessment of pubertal development followed international standards, defining adolescence as 13–15 years for males, and 12–13 years for females (10).

Immunosuppressive therapy: The immunosuppressive protocol consisted of induction therapy with either anti-CD25 monoclonal antibody or rabbit anti-thymocyte globulin, followed by corticosteroid withdrawal within the first postoperative week. The maintenance treatment was tacrolimus combined with mycophenol sodium enteric-coated tablets combined with immunosuppressive therapy. Target tacrolimus trough levels were maintained at 10–15 ng/ml during the first month, 8–10 ng/ml for the initial three months, and 5–8 ng/ml thereafter. Mycophenolate sodium was administered at a dose of 540 mg·1.73 m^−^²·d^−^¹.

Delayed graft function (DGF) was defined as the requirement for dialysis within the first week post-transplantation (11). Rejection episodes were characterized by clinical manifestations including elevated serum creatinine levels or proteinuria, confirmed through allograft biopsy as either antibody-mediated rejection (AMR) or T cell-mediated rejection (TCMR) (12).

Statistical analysis: Statistical analyses were performed using SPSS software (version 22.0). Normally distributed continuous variables were expressed as mean ± standard deviation, while non-normally distributed data were presented as median. Comparisons between groups were conducted using Student's *t*-test for continuous variables and *χ*^2^ test for categorical variables, with *P* < 0.05 considered statistically significant. Graft and recipient survival rates were calculated using the Kaplan–Meier method.

## 3 Results

### 3.1 Patient characteristics

All kidney transplants in our center were from deceased donors. There were 85 donors in this survey (Table 1), with an average age of 37.8 months, a median age of 13 months (0.3–192), an average weight of 14.1 kg, and a median weight of 10 kg (3.2–68). The most common cause of death was head trauma; 36 cases (accounting for 47.4%), followed by congenital hypoplasia; 21 cases (accounting for 27.6%), then malignant brain tumor; 9 cases (accounting for 11.8%), asphyxia: 6 cases (accounting for 7.9%), severe infection: 5 cases (accounting for 6.6%), and 8 cases of unknown reason of death (accounting for 10.5%). This study enrolled 101 patients, comprising 56 males and 45 females. Preoperatively (Table 1), 79 patients (78.22%) underwent peritoneal dialysis, 17 patients (16.83%) received hemodialysis, and 5 patients (4.95%) had no dialysis history. The median dialysis duration was 14 months (range: 0–88 months). At transplantation, the median age was 11.4 years (range: 2–17.6 years), with a median follow-up age of 18 years (range: 6–26 years) and a median post-transplant follow-up duration of 80 months (range: 30–138 months). The transplantation modalities included 2 cases of combined liver-kidney transplantation and 10 cases of *en bloc* kidney transplantation (Postoperative thrombosis occurred in 3 cases and secondary single kidney transplantation was performed after resection), along with 89 cases of single kidney transplantation. The most prevalent etiology of ESKD (Table 2) was chronic glomerulonephritis (44 cases, 43.56%), followed by immune-mediated nephropathy (21 cases, 20.79%), and congenital anomalies of the kidney and urinary tract (15 cases, 14.85%). Other causes included polycystic kidney disease (5 cases, 4.95%), genetic mutations (5 cases, 4.95%), primary hyperoxaluria (2 cases, 1.98%), hypertensive nephropathy (1 case, 0.99%), and unknown etiologies (8 cases, 7.92%). All donor-recipient pairs were ABO blood type identical or compatible, with negative panel reactive antibodies in recipients and negative lymphocyte crossmatch results in all cases.

**Table 1:** Characteristics of kidney transplant donors and recipients.

Variables		Total
Donor characteristics	Age(months)	13 (0.3–192)
	Weight (kg)	10 (3.2–68)
Cause of death	Head trauma	36 (47.4%)
	Congenital hypoplasia	21 (27.6%)
	Malignant brain tumor	9 (11.8%)
	Asphyxia	6 (7.9%)
	Severe infection	5 (6.6%)
	Unknown reason	8 (10.5%)
Recipients characteristics	Number of cases (male/female)	101 (56/45)
	Median dialysis time	14 months (0–88)
	Median age at transplantation	11.4 years (2–17.6 years)
	Median age at follow-up	18 years (6–26 years)
Mode of dialysis	PD	79
	HD	17
	Not dialysed	5
Transplantation methods	Combined liver and kidney transplantation	2 (1.98%)
	En bloc double kidney transplantation	10 (9.9%)
	Single kidney transplantation	89 (88.12%)

**Table 2:** The causes of end-stage kidney disease.

Primary diseases	Chronic nephritis	44 (43.56%)
	Immune nephropathy	21 (20.79%)
	Congenital hypoplasia	15 (14.85%)
	Polycystic kidney	5 (4.95%)
	Genetic mutations	5 (4.95%)
	Primary hyperoxaluria	2 (1.98%)
	Hypertensive nephropathy	1 (0.99%)
	Unknown cause	8 (7.92%)

### 3.2 Recipient and graft survival

**Figure 1:**
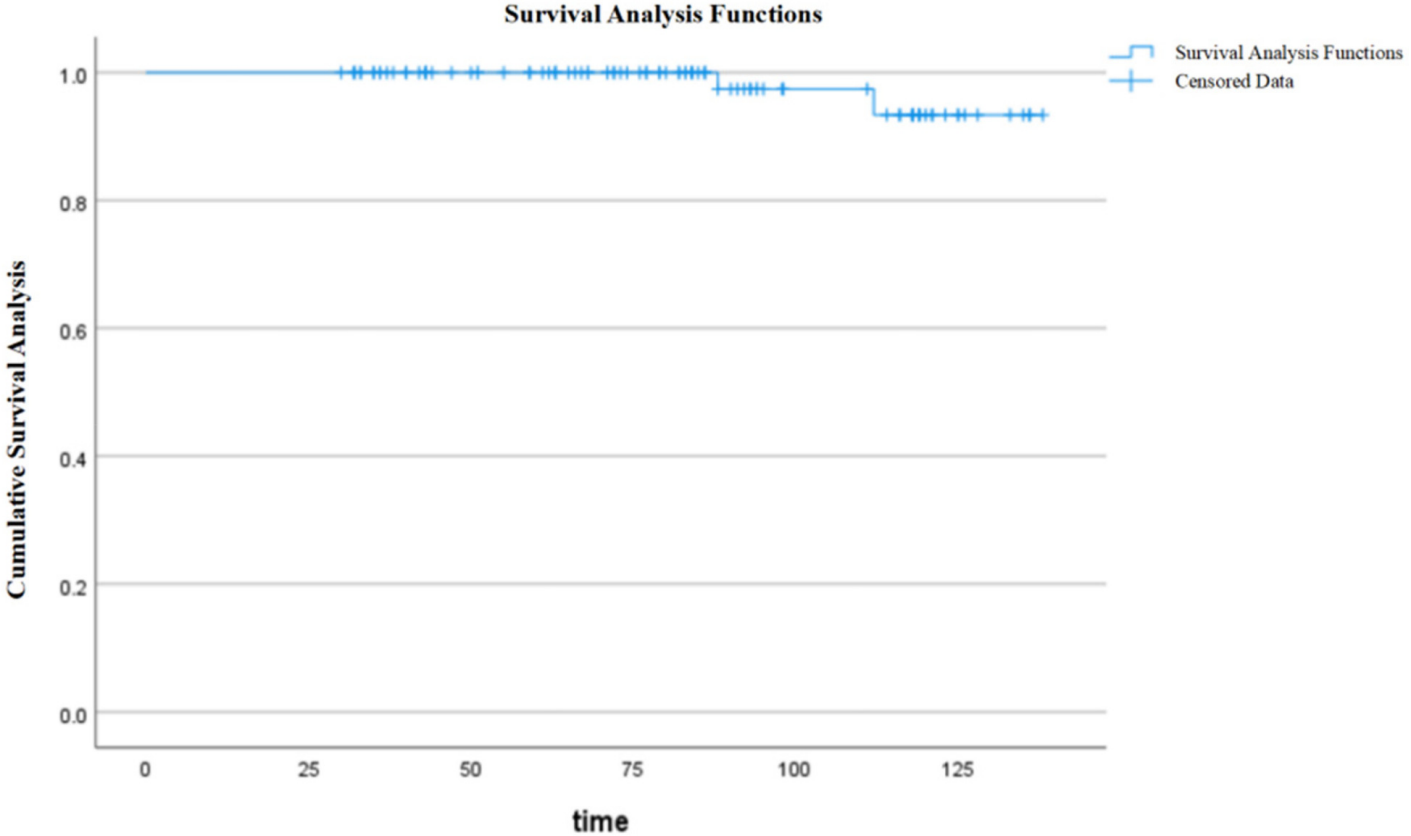
Graft survival curves.

During follow-up, graft function remained stable, with a mean serum creatinine level of 83.85 ± 38.34 μmol/L and an eGFR of 79.49 ± 27.25 ml/min/1.73 m^2^ at the last follow-up. Among the study cohort, 74 patients had a transplant duration exceeding 5 years. The graft survival rates were 100% and 93.4% at 5 and 10 years, respectively (Figure 1), while patient survival rates remained 100% at both time points. Among the study cohort, 91 patients received single kidney transplants while 10 underwent *en bloc* kidney transplantation. Notably, two recipients in the *en bloc* kidney transplantation group developed graft thrombosis postoperatively, necessitating transplant nephrectomy, but both patients subsequently underwent successful single kidney retransplantation. Post-transplant complications were observed in 45 cases, including infections (pulmonary and urinary tract): 43 cases; acute rejection: 24 cases; post-transplant lymphoproliferative disorder (PTLD): 2 cases; delayed graft function (DGF): 17 cases; renal artery stenosis: 7 cases; cytomegalovirus (CMV) infection: 3 cases; parvovirus B19 infection: 3 cases; and ureteral stenosis: 1 case. During the follow-up period, two cases of graft loss and blood dialysis recovery were observed.

### 3.3 Post-transplant growth

Post-transplant growth outcomes are presented in (Table 3). Significant improvement in growth parameters was observed, with the proportion of children exhibiting growth delay (HAZ < −2) decreasing from 52.48% pre-transplantation to 15.84% at final follow-up (*P* < 0.001). Among all pediatric recipients, 85 cases (84.16%) achieved near-final height (NFH) within the normal range for their age group (Table 4a), including 46 males (54.12%) with a median NFH of 166 cm (range: 115–183 cm) and 39 females (45.88%) with a median NFH of 158 cm (range: 143–171 cm). Sixteen recipients (15.84%) demonstrated significantly lower NFH compared to their age-matched peers, comprising 10 males (62.5%) with a median NFH of 155 cm (range: 151–161 cm) and 6 females (37.5%) with a median NFH of 149.5 cm (range: 140–150 cm) (*P* > 0.05). All 16 recipients with subnormal NFH had pre-transplant heights below the normal range, and except for 4 recipients aged <12 years, the remaining 12 were >12 years old. The younger the age at transplantation, the higher the proportion of patients reaching the standard height (|Z|≤2) at follow-up (Table 4b), while there was no difference in those that achieved standard height by sex. Statistical analysis revealed that NFH in pediatric transplant recipients correlated significantly with age at transplantation and height at transplantation (*P* < 0.05), but showed no association with gender, dialysis modality, or dialysis duration (*P* > 0.05).

**Table 3:** Comparison of HAZ values at the time of surgery and follow-up (*n* = 101).

Item	Height at transplantation			Height at last follow-up		
	Male	Female	Total	Male	Female	Total
HAZ value	−2.31 ± 1.52	−2.21 ± 1.78	−2.27 ± 1.64	−0.74 ± 1.26	−0.79 ± 0.94	−0.76 ± 1.13
HAZ < −2 (%)	32	21	53 (52.48%)	10	6	16 (15.84%)
−2 < HAZ < 0 (%)	19	19	38 (37.62%)	27	33	60 (59.41%)
HAZ > 0 (%)	5	5	10 (9.9%)	19	6	25 (24.75%)

**Table 4a:** Height at follow-up (*n* = 101).

Item	Height at follow-up		
	Male	Female	Total
Reaching standard height (median)	46 (54.12%)	39 (45.88)	85
	166 cm (115 cm–183 cm)	158 cm (143 cm–171 cm)	162 cm (115 cm–183 cm)
Not reaching standard height (median)	10 (62.5%)	6 (37.5%)	16
	155 cm (151 cm–161 cm)	149.5 cm (140 cm–150 cm)	150 cm (140 cm–161 cm)

**Table 4b:** Height at follow-up at different ages at transplant (*n* = 101).

Age group	Item	Height at follow-up		
		Male	Female	Total
2–12 (years)	Reaching standard height (median)	31	23	54
		166 cm (115 cm–183 cm)	158 cm (145 cm–170.5 cm)	161 cm (115 cm–183 cm)
	Not reaching standard height (median)	3	1	4
		150 cm (140 cm–150 cm)	143 cm	146.5 cm (140 cm–150 cm)
12–18 (years)	Reaching standard height (median)	15	16	31
		165 cm (162 cm–173.5 cm)	158 cm (15 cm–165 cm)	162 cm (153 cm–173.5 cm)
	Not reaching standard height (median)	7	5	12
		157.5 cm (151 cm–161 cm)	150 cm (142 cm–150 cm)	153 cm (142 cm–161 cm)

### 3.4 Pubertal development status

The study cohort comprised 56 male patients, including 43 (76.8%) who had attained pubertal onset (age >13 years). Among these pubertal patients, 37 (86.0%) achieved spermarche, with 4 cases occurring prior to kidney transplantation and 33 cases developing postoperatively. The mean age at spermarche was 14.5 years in prepubertal transplant recipients and 15 years in postpubertal recipients (*P* > 0.05). The cohort included 45 female patients, with 42 (93.3%) having reached puberty (age >12 years). Among pubertal patients, those who underwent kidney transplantation before puberty had a mean menarche age of 12.5 years, whereas those transplanted after puberty showed a mean menarche age of 13.2 years (*P* > 0.05). Of these, 7 patients experienced menarche prior to kidney transplantation surgery, while 35 cases occurred postoperatively. Menstrual irregularities were observed in 8 patients (19.5% of pubertal individuals).

### 3.5 Educational attainment and employment status

**Figure 2:**
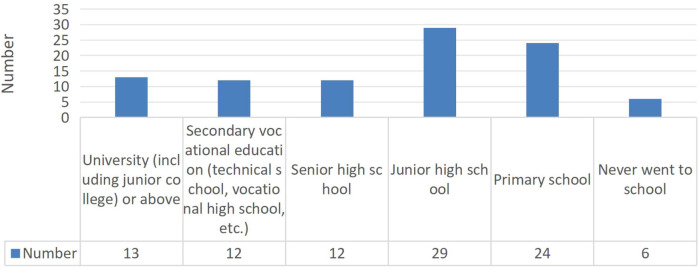
Level of education.

Among 96 school-aged patients in the follow-up cohort (Figure 2), 90 (93.75%) were actively pursuing their education. Employment outcomes were suboptimal, with only 33.3% of recipients who had completed their education being employed, though this analysis was limited by the small number of patients who had finished their academic pursuits.

## 4 Discussion

Kidney transplantation represents the optimal treatment for children with end-stage renal disease, offering benefits beyond renal function improvement. For pediatric recipients, transplantation not only promotes physical growth and pubertal development but also significantly contributes to psychological well-being and cognitive development, enabling better engagement in daily activities and social participation (13, 14). Prolonged dialysis in children with CKD can lead to endocrine dysfunction and metabolic disturbances, resulting in adverse outcomes including growth delay and delayed pubertal development (15). Prolonged dialysis in children with CKD may also lead to psychological complications and psychosocial challenges, potentially causing emotional disturbances and significantly impacting educational attainment and social interactions (16). Although these children with CKD are well cared for by their families and society, long-term personal and social problems still exist (17, 18).

The severity of growth delay in pediatric patients shows a strong inverse correlation with the age at CKD onset, with earlier disease manifestation associated with more pronounced growth impairment (17). Japanese studies evaluating long-term outcomes of pediatric kidney transplantation recipients have identified persistent short stature as a significant concern, with approximately 30% of patients achieving a final height below −2.5 standard deviation scores (SDS) 2. Consistent with our findings, most pediatric patients exhibited short stature prior to kidney transplantation, falling below the normal range for their age group (19, 20). In this study, 52.48% of recipients demonstrated growth delay (HAZ < −2) pre-transplantation, which significantly decreased to 15.84% at final follow-up (*P* < 0.001). Height at the start of dialysis and duration of dialysis are important determinants of baseline height at renal transplant (21). Our study revealed that longer pre-transplant dialysis duration correlated with lower height Z-scores, with approximately 15.84% of recipients achieving near-final height (NFH) below that of their healthy peers. Height at dialysis initiation and age at dialysis onset were identified as significant determinants of NFH, highlighting the importance of early transplantation, including pre-emptive transplantation, to minimize dialysis duration and its detrimental effects on growth (22–24). However, neither dialysis modality nor dialysis duration showed significant impact on final NFH (*P* > 0.05).

Another significant complication in children with CKD involves pubertal development and sexual maturation disorders. For pre-dialysis patients, management primarily focuses on preserving renal function and implementing growth hormone therapy, while therapeutic options for improving pubertal development remain limited (15). Research indicates that maintaining optimal pubertal development and normal sexual maturation presents a significant clinical challenge in managing children with CKD: approximately 50% of patients requiring renal replacement therapy (RRT) before age 13 exhibit delayed pubertal development, while early kidney transplantation represents the most effective intervention for improving pubertal progression and sexual maturation in pediatric CKD patients (25). Ferraris et al. (26) demonstrated that following successful kidney transplantation, nearly all patients demonstrated serum testosterone levels within the normal range for their pubertal stage, and follicle-stimulating hormone (FSH) levels were normal in two-thirds of the recipients. Winkler et al. (27) observed that following successful kidney transplantation female recipients exhibited normal cyclic fluctuations of gonadotropins and estradiol (E2) corresponding to ovulatory cycles. Our findings suggest that most pediatric patients can achieve normal pubertal development and sexual maturation following successful kidney transplantation. However, studies indicate that pubertal growth (particularly in pre-pubertal kidney transplantation recipients) and sexual maturation are associated with post-transplant immunosuppressive regimens, highlighting the need for more effective immunosuppressive protocols to facilitate normal physical and sexual development in children with growth and maturation delays (28).

Pediatric kidney transplantation enables timely reintegration into school and society, allowing school-aged children to pursue normal education and social development. Studies report that most kidney transplantation recipients successfully continue their education, and the majority of adult patients secure appropriate employment (2). Although some children experience grade-age mismatch or lower employment rates compared to the general population, they perceive their quality of life as comparable to their peers (14). In our study cohort, 96 school-aged children were identified, with 90 (93.75%) actively pursuing their education, demonstrating successful school reintegration. However, the employment rate was only 33.3% among the limited number of adult patients available for assessment. According to statistics released by China's National Bureau of Statistics, the employment rate among healthy individuals aged 16–24 years in 2024 was approximately 84.3%. The lower employment rate observed in kidney transplant recipients may be attributed to the following potential contributors: (1) this study's single-center retrospective design and limited sample size; (2) a majority of followed patients were still pursuing education; (3) patients' and families' concerns about employment interfering with medication adherence and follow-up care; (4) employers' reservations regarding hiring kidney transplant recipients; and (5) the limited availability of suitable job opportunities for this population at the societal level.

As pediatric kidney transplant recipients age and undergo physiological maturation, various factors encountered during this developmental process may potentially impact graft function, potentially leading to graft failure (6). During adolescence, the progressive maturation of the immune system may heighten immunological responses against the allograft, particularly increasing the risk of T cell-mediated rejection (TCMR) and antibody-mediated rejection (ABMR). This heightened HLA antibody production risk may be associated with suboptimal immunosuppression or poor medication adherence. Medication adherence is particularly crucial for adolescent kidney transplant recipients (29), but there can be difficulty with medication adherence in adolescent kidney transplant recipients due to psychosocial factors, limited disease awareness, social pressures, and mental health issues. Additionally, medication side effects such as acne and hirsutism that affect physical appearance may contribute to missed doses or self-initiated dose reduction or discontinuation of immunosuppressive therapy. Adolescent patients may experience interrupted follow-up and inadequate monitoring due to educational or occupational commitments, potentially leading to delayed recognition and management of rejection episodes or complications, particularly when facing changes in healthcare locations or transplant team transitions. We observed two patients with graft failure and resumed hemodialysis during follow-up. One patient always forgot to take medication for work reasons, and the other patient went to the hospital for examination and found that creatinine had irreversibly increased due to non-adherence to follow-up appointments. This study identified non-adherence as the primary contributor to rejection episodes and graft loss, manifested as self-discontinuation of immunosuppressive medications or prolonged loss to follow-up without medical consultation. Studies have shown that while many individuals successfully adapt to post-transplant life, achieving psychological and emotional growth with life satisfaction comparable to healthy peers—often demonstrating increased motivation and energy—a subset of patients continue to perceive themselves as chronically ill, experiencing difficulties with concentration, learning challenges, and episodes of anxiety and depression (14). Therefore, adolescent patients require comprehensive psychological support from both their families and healthcare teams to prevent treatment non-adherence resulting from depression, anxiety, or rebellious behaviors.

## 5 Conclusions

This study demonstrates significant improvements in height attainment, pubertal development, and social outcomes following pediatric kidney transplantation. While recipient gender, pre-transplant dialysis modality, and dialysis duration showed no significant impact on NFH, both age at transplantation and height at transplantation significantly influenced NFH. These findings emphasize that early transplantation and maintaining optimal graft function are crucial for ameliorating growth delay and pubertal development, while also positively influencing long-term social outcomes in pediatric transplant recipients.

## 6 Limitation

This single-center retrospective study has inherent limitations including a modest sample size and insufficient follow-up duration, with the majority of participants remaining school-aged at last assessment. While our analysis demonstrates that early kidney transplantation confers physical and psychological benefits for children with ESKD, crucial long-term social outcomes—particularly employment status, marital prospects, and reproductive health—remain unassessed due to current data constraints. To comprehensively evaluate the longitudinal impacts of pediatric renal transplantation, future multi-center collaborative studies with extended follow-up periods are imperative. Such investigations will better elucidate the lifelong benefits of transplantation in this vulnerable population while addressing critical knowledge gaps in post-transition care.

## Data Availability

The original contributions presented in the study are included in the article/Supplementary Material, further inquiries can be directed to the corresponding author/s.
